# Evaluating the phylogenetic signal of morphosyntax

**DOI:** 10.1515/psicl-2025-0030

**Published:** 2026-02-09

**Authors:** Ruby Sleeman, Maria-Margarita Makri, Elena Anagnostopoulou, Emmanuel D. Ladoukakis, Dimitris Michelioudakis, Christos Zioutis, Pavlos Pavlidis

**Affiliations:** Institute for Mediterranean Studies – Foundation for Research & Technology Hellas (IMS-FORTH), Heraklion, Greece; Department of Philology, Division of Linguistics, University of Crete, Rethymnon, Greece; Department of Biology, University of Crete, Heraklion, Greece; Department of Linguistics, School of Philology, Aristotle University of Thessaloniki, Thessaloniki, Greece; FORTH-Institute of Computer Science (FORTH-ICS), Heraklion, Greece

**Keywords:** phylogenetics, historical linguistics, linguistic typology, Indo-European, morphosyntactic variation

## Abstract

Computational linguistic phylogenetics has so far relied heavily on cognate data. In contrast, the potential of morphosyntactic characters as a valuable source for phylogenetic analysis has been largely overlooked. We argue that morphosyntactic characters may conflate historical signal with the results of homoplasies, horizontal transfer, and universal tendencies, and must be scrutinized in terms of their propensity to change and borrowing, analogously to the curation of lexical data which produced the Swadesh lists. In this paper we make a start by evaluating a set of morphosyntactic characters based on the World Atlas of Language Structures using three methods: we (1) calculated Pearson correlation coefficients for each character against different language groupings, reflecting either shared ancestry (genera) or contact (geographical proximity); (2) counted the minimum number of mutations needed for the distribution of a character’s states on a cognate-based reference tree (parsimony score), testing whether they correctly reflect language change known from historical linguistics; and (3) ran a classic hill-climbing algorithm to determine which random subsets of characters produced a phylogeny closest to a reference tree. We conclude that these are useful tools, but expect that making the definitions of the characters more theoretically informed will produce a stronger historical signal.

## 1 Introduction: studying morphosyntax via phylogenies

Shared traits between languages can have four potential sources: a shared trait may have been inherited from a common ancestor; it could be the result of horizontal transfer between the languages through contact; the trait could have developed independently according to some trend common to human language in general; and lastly, there is always a chance of a coincidental commonality. In practice, these four phenomena, inheritance, horizontal transfer, convergence with universal trends, and homoplasy, are not trivially teased apart. A fairly new field has arisen in the last couple of decades on an endeavor to disentangle linguistic evolution by means of tracking inheritance: computational phylogenetics in linguistics (see Ladoukakis et al. 2022 and references therein).

The common practice for this young and upcoming field has been to build phylogenies on sets of cognate data. The current paper seeks to contribute to this growing field by asking the question: To what extent can *morphosyntactic* data be exploited to determine common ancestry? We describe three computational methods that we employed with the aim to scrutinize morphosyntactic characters based on the World Atlas of Language Structures (WALS, Dryer and Haspelmath 2013), in terms of their potential to carry phylogenetic signal. We focused our efforts on the Indo-European (IE) language family, because there is a plethora of written historical data for this family, thus allowing us to compare the potential signal of our morphosyntactic data against a given reference phylogeny, built upon fairly uncontroversial, established data concerning cognate lexical items. We elaborate on this reference tree below.

Computational linguistic phylogenetics has so far relied heavily on cognate data. These have produced, by means of Bayesian inference, phylogenies largely aligning with existing knowledge of language history (Bouckaert et al. 2012; Chang et al. 2015; Sagart et al. 2019; a.o.). But linguistic variation, and commonalities between languages, go beyond their lexical inventories: languages may have morphemes in common, they may show similar developments, e.g. loss of cases, syncretism across or within paradigms, changes in word order preferences, the innovation of a set of definite articles where a previous stage had none. Moreover, looking at lexical items that share an etymology can only take the researcher as far back as the earliest attestation, or in some cases, a reconstructed previous stage, if there is enough evidence to extrapolate.

The idea that morphosyntax has the potential to track deeper historical and geographical relationships, and may therefore be of value to the linguistic phylogenetic enterprise, has been around for some time (Greenhill 2021; Matsumae et al. 2021; Nichols 1992; Santos et al. 2020), and some attempts have been undertaken to build phylogenies on the basis of morphosyntactic characters: Dunn et al. (2008); Dunn et al. (2005); and Wichmann and Saunders (2007) are examples, but none of these papers focus on the phylogenetic signal of the features used. Within generative linguistics, Longobardi and Guardiano (2009) proposed to build linguistic phylogenies using syntactic parameters; their ‘Parametric Comparison Method’ focuses on the nominal domain and has applied distance-based methods to build phylogenetic trees for a number of Indo-European languages and other language groups (Guardiano and Longobardi 2017; Longobardi and Guardiano 2009). Hartmann and Walkden (2024) also discuss the importance of measuring phylogenetic signal, but they choose to measure this by specifically using non-curated data:

We use [the SSWL] dataset [(Koopman 2012 as cited by Hartmann and Walkden 2024)] *specifically because it reflects raw syntactic properties* [our emphasis] and was not designed to be used in phylogenetic analyses. Our aim in doing so is to assess the extent to which syntactic variation contains phylogenetic signal (Hartmann and Walkden 2024).

Although we do agree with these accounts that morphosyntactic characters have the potential to provide a source for constructing phylogenies, we believe that a crucial first step is to scrutinize such characters in terms of their propensity to change and borrowing, analogously to the curation of lexical data that has led to the creation of the sets of vocabulary that are more likely to resist borrowing cross-linguistically, commonly known as the Swadesh lists (Swadesh 1952, 1955). Without this kind of scrutiny, phylogenies based on raw morphosyntactic data are bound to carry a mixed signal: whenever multiple languages that are historically related to each other happen to share an innovation or a retention, it is not a given that that change was necessarily inherited; nonetheless, computational phylogenetic methods typically assume exactly this.

Thus, we argue that without properly scrutinizing morphosyntactic characters, and simply running a Bayesian phylogenetic inference on existing datasets such as SSWL or WALS, the resulting trees will reflect a mixture of genealogical, geographical, and typological signal. In addition, questions regarding the stability of morphosyntactic characters on different taxonomic levels, on their propensity to change and borrowing, are genuine questions that regard the nature of the data, not just their usefulness for reconstructing history.

We need to scrutinize morphosyntactic data in order to find out:

- Which morphosyntactic characters are most fitting and adequate for detecting vertical transmission;
- Whether it is possible to identify a large pool of suitable morphosyntactic characters (analogous to the Swadesh lists), or if relativizations to areas and families are required;
- Whether it is individual morphosyntactic characters that produce reliable phylogenies, or rather bundles of characters that covary.

To make a start on answering these questions, we evaluated our dataset (which we describe in more detail below) using three methods: (1) We calculated Pearson correlation coefficients for each character, thus testing their support of bipartitions in the IE tree, reflecting either shared ancestry (genera) or contact (geographical proximity); (2) We counted the number of mutations (parsimony score) for the distribution of a character’s states on a cognate-based reference tree, to test whether they correctly reflected known historical events of language change; and lastly, (3) a classic hill-climbing algorithm to determine which randomly picked subsets of characters produced a phylogeny closest to a reference cognate-based tree.

The remainder of this paper is structured as follows: in Section 2 we describe the language sample and the dataset we used to evaluate the morphosyntactic characters; in Sections 3, 4, and 5 we present the three methods along with their results; Section 6 concludes the paper.

## 2 Materials

### 2.1 The language sample

We conducted three studies on data drawn from 46 old and modern languages representing most IE genera, for which we collected morphosyntactic data and for which cognate data was available (Bouckaert et al. 2012). We now list the genera followed by the languages in round brackets: Albanian (Standard Albanian), Armenic (Eastern Armenian), Baltic (Latvian, Lithuanian), Slavic (Belarusian, Bulgarian, Macedonian, Old Church Slavonic, Polish, Russian, Serbo-Croatian, Slovak, Slovenian, Ukrainian), Celtic (Breton, Irish, Scottish Gaelic, Welsh), Germanic (Danish, Dutch, English, Faroese, German, Gothic, Icelandic, Old English, Swedish), Greek (Classical Greek, Standard Modern Greek), Indo-Iranian (Bengali, Gujarati, Hindi, Kashmiri, Marathi, Nepali (Gurkhali), Pashto, Persian, Sanskrit), and Italic (Catalan, European Portuguese, French, Italian, Latin, Romanian, Spanish).

### 2.2 Morphosyntactic data

The morphosyntactic dataset was based on the World Atlas of Language Structures (WALS, Dryer and Haspelmath 2013). For the 46 languages listed above, we collected data for features falling in the following categories: Morphology (12), Nominal Categories (28), Nominal Syntax (8), Verbal Categories (17), Word Order (56), Simple Clauses (24), Complex Sentences (7), Lexicon (4), following the sampling procedures described in the respective WALS chapters. Features that had more than 25% of missing values were excluded. The remaining features fell in the following categories: Morphology (8), Nominal Categories (23), Nominal Syntax (7), Verbal Categories (13), Word Order (20), Simple Clauses (18), Complex Sentences (1), Lexicon (2). Most of the features excluded were non-applicable in IE (e.g. a big number of word order features could only be defined under conditions absent in all IE languages) or features for which it was difficult to find precise data, due to the fuzziness of the definition (e.g. 22A *Inflectional synthesis of the verb*). Then, the resulting table was binarized by converting each WALS state to a binary character, where value 1 was assigned if a language had the state in question and value 0 if not. For example, feature 21A *Exponence of selected inflectional formatives* was converted to five binary characters: (1) Does the language have monoexponential case (Y/N), (2) Does the language have fusion of case and number (Y/N), (3) Does the language have fusion of case and referentiality (Y/N), (4) Does the language have fusion of case and TAM (Y/N), (5) Does the language lack exponence of case (Y/N)? The final dataset consisted of 425 binary characters with at least 75% coverage for the 46-language sample. The full list of characters can be found in the Supplementary material.1Because we turned WALS states into characters, we have a code for each of these binarized states. In the file ‘Characters_to_WALS_states’, our characters are mapped to their corresponding WALS chapters and values.


## 3 Bipartition-character correlations

A bifurcating phylogenetic tree can be cut into two portions at any node, thus dividing the whole tree into two subgroups. This is a bipartition of the tree. This information can be alternatively represented as assigning value 1 to languages of the one subgroup and 0 for the other. For example, suppose that the two groups we examine are the Germanic languages on the one hand (all the languages belonging to that group in the tree receive the value 1) and all the other languages on the other hand (receiving the value 0). With this process we have produced a column containing the value 1 for the Germanic languages and the value 0 for the rest of the languages. This represents the bifurcation of the tree. Then, we create a second column containing the value 0 or 1 for a binary character for all languages of the tree. For these two columns we calculated the Pearson Correlation Coefficient (PCC). High absolute values of correlation coefficient suggest that the feature under examination supports the specific bipartition of the tree. If we repeat this procedure for every feature we can identify all the features that support the specific node of the tree, in this example we can identify which features support the grouping of Germanic languages into a monophyletic group in the tree. Therefore, this information is genealogical information. Then we can apply the same procedure to any language group that has a meaning for us.2This method, in principle, does not need an actual tree to work upon. It can also work by artificially stipulating groups of languages (genealogical, geographical or any other group) that meet a certain criterion (e.g. historical, geographical or typological) and thus offer a hypothesis to test. Then we can ask which characters support this grouping.


Let’s assume that in our tree the languages of the Balkan Sprachbund are grouped together and in a single node of the tree they are separated from the rest of the tree. If we repeat the previous procedure, we can identify characters that support the grouping of the Balkan Sprachbund languages. Given that this group is polyphyletic and its languages share geography rather than genealogy, such characters will contain areal and not genealogical information. We calculated correlations between all our characters and the language groups shown in Table 1.

**Table 1:** Language groups tested. H = Historically related (vertical transmission of shared traits), G = Geographically close (horizontal transmission of shared traits).

Language group	Languages included in the group	H/G
**Italic**	Italian, Spanish, Catalan, French, European Portuguese, Romanian, Latin	H
**Romance**	Italian, Spanish, Catalan, French, European Portuguese, Romanian	H
**Celtic**	Irish, Welsh, Scottish Gaelic, Breton	H
**Balto-Slavic**	Bulgarian, Macedonian, Serbo-Croatian, Slovak, Old Church Slavonic, Czech, Slovak, Polish, Russian, Ukrainian, Belarusian, Latvian, Lithuanian	H
**Modern Balto-Slavic**	Bulgarian, Macedonian, Serbo-Croatian, Slovak, Czech, Slovak, Polish, Russian, Ukrainian, Belarusian, Latvian, Lithuanian	H
**Slavic**	Bulgarian, Macedonian, Serbo-Croatian, Slovak, Old Church Slavonic, Czech, Slovak, Polish, Russian, Ukrainian, Belarusian	H
**Baltic**	Latvian, Lithuanian	H
**Modern Slavic**	Bulgarian, Macedonian, Serbo-Croatian, Slovak, Czech, Slovak, Polish, Russian, Ukrainian, Belarusian	H
**Macedonian-Bulgarian**	Bulgarian, Macedonian	H
**Germanic**	Gothic, Old English, English, German, Dutch, Danish, Icelandic, Swedish, Faroese	H
**Modern West Germanic**	English, German, Dutch	H
**North Germanic**	Danish, Icelandic, Swedish, Faroese	H
**Modern Germanic**	English, German, Dutch, Danish, Icelandic, Swedish, Faroese	H
**Indo-Iranian**	Sanskrit, Marathi, Hindi, Gujarati, Kashmiri, Bengali, Nepali, Farsi, Pashto	H
**Modern Indo-Iranian**	Marathi, Hindi, Gujarati, Kashmiri, Bengali, Nepali, Farsi, Pashto	H
**Indic**	Sanskrit, Marathi, Hindi, Gujarati, Kashmiri, Bengali, Nepali	H
**Iranian**	Farsi, Pashto	H
**Modern Indic**	Marathi, Hindi, Gujarati, Kashmiri, Bengali, Nepali	H
**Albanian**	Albanian^a^	H
**Armenian**	Armenian	H
**Greek**	Classical Greek, Greek	H
**Modern Greek**	Greek	H
**Balkan**	Albanian, Romanian, Bulgarian, Macedonian	G
**Albanian-Romanian**	Albanian, Romanian	G

^a^Some of these ‘language groups’ correspond to genera in the IE family. For this reason, the groups Albanian, Armenian, and Modern Greek include only one language. Other genera happen to be comprised of more languages; however, a singleton set is still considered a set.

The resulting correlation coefficient values (r) are interpreted as follows: if a character has a correlation value close to 1 with a given language group, it means that its *presence* is characteristic of this group within the language sample, in our case IE; if r is close to −1 it means that the *absence* of this character (WALS feature state) is characteristic of this group within IE; while an r value close to 0 means the absence of a correlation. If there is a correlation between a character and a genealogical group (e.g. genus) we take this to mean that a character carries phylogenetic signal, while a correlation between a character and a group of languages not genealogically connected may indicate that the character is prone to horizontal transfers or that there have been abrupt changes and simplifications due to intense contact and L2 influence (Trudgill 2011).3All languages in our sample belong to the same family so there is some genealogical connection. For the purposes of these tests the level of comparison is genus: the languages in the Balkan Sprachbund belong to different genera. The notion of ‘genus’ as a term for a subfamily is motivated by Dryer (1989: 267); he draws a comparison to “the taxonomic level of genus in biology”.


The correlation values were analyzed in the following ways: First (method (a)), for each language group tested, the characters were sorted based on the r value obtained. Then, we examined the list of characters and searched for a correlation value that could function as a threshold between characters that reflect typical properties of the language group in question based on existing literature, and characters that do not. This process was repeated for each language group. We accepted as informative characters that had correlation coefficients higher than r ≈ 0.30 for positive correlations and lower than r ≈ −0.30 for negative correlations (or, alternatively, an absolute value of |r| ≈ 0.30 was set as a threshold). These correlation values might appear low but we accepted them after evaluation of our results with qualitative, linguistically informative methods (method (a)). The full list of correlation values per group per character along with descriptions of the characters can be found in the Supplementary material.4‘Bipartitions_Pearson_correlation_values_for_all_groups_and_characters’.


In what follows we take a closer look at the characters that obtain the highest absolute correlation values in a given genus. Table 2A illustrates the characters with the highest positive correlation values for the Indo-Iranian genus and Table 2B the ones with the lowest negative values. These characters reflect properties of Indic and/or Iranian independently observed in the literature: clause-final position of the verb (Hale 2018; Masica 1991: 332–333), postpositions (Masica 1991: 333), prenominal adjectives (Masica 1991: 370; Taghipour and Kehnemuyipour 2023), reduplication (Masica 1991: 78–79; Sims-Williams 1993), wh-in-situ (Hale 2018; Masica 1991: 386), and monoexponential case (Kulikov 2017: 211); the negative correlations are also compatible with properties not found in Indo-Iranian.5NB the prenominal adjective per se is not characteristic for Indo-Iranian, only its combination with post-verbal objects.
^,^ 6A comparison between the characters with negative correlation values and positive correlation values shows that, for any given branch, the characters with strong negative correlations most of the times belong to WALS features also found in the list of characters with strong positive correlations; this is an artifact of creating characters by binarizing WALS features. This shows us that the method indeed helps identify characters for which their presence or absence is (a)typical of a given language group.

**Table 2A:** Strong positive Pearson Correlation Coefficients for the Indo-Iranian genus.

Character	Description (WALS chapter)	PCC
81A1	SOV (Order of Subject, Object and Verb)	0.86
83A1	OV (Order of Object and Verb)	0.86
93A2	Not initial interrogative phrase (Position of Interrogative Phrases in Content Questions)	0.80
21A1	Monoexponential case (Exponence of Selected Inflectional Formatives)	0.71
27A1	Productive full and partial reduplication (Reduplication)	0.79
95A1	OV and Postpositions (Relationship between the Order of Object and Verb and the Order of Adposition and Noun Phrase)	0.86
97A1	OV and AdjN (Relationship between the Order of Object and Verb and the Order of Adjective and Noun)	0.79
85A1	Postpositions (Order of Adposition and Noun Phrase)	0.86
43A2	Related for all demonstratives (Third Person Pronouns and Demonstratives)	0.71

**Table 2B:** Strong negative Pearson Correlation Coefficients for the Indo-Iranian genus.

Character	Description (WALS chapter)	PCC
93A1	Initial interrogative phrase (Position of Interrogative Phrases in Content Questions)	−0.88
85A2	Prepositions (Order of Adposition and Noun Phrase)	−0.86
27A3	No productive reduplication (Reduplication)	−0.75
106A2	Mixed (Reciprocal Constructions)	−0.75
53A3	First, second, three-th (Ordinal Numerals)	−0.71
99A1	Nominative – accusative (standard) (Alignment of Case Marking of Pronouns)	−0.71

We can then turn to another example, that of a language group composed of languages belonging to different genera of IE but forming a Sprachbund. Some well-known Balkanisms are (i) Postpositive articles, (ii) the loss of infinitives and their replacement by subjunctive clauses and (iii) clitic doubling of objects (Tomić 2006 and references cited therein). Table 3 shows the characters with the highest correlation values with the Balkan language group: indeed, the Balkanisms identified in the literature correspond to the characters with the highest r values, more specifically, (i) above maps to 37A3, (ii) to 124A2 and (iii) to 102A3, 104A3, and 104A4.7Our WALS-based database did not contain information for other Balkanisms identified in the literature, e.g. case marking by suffix or preposition, dative clitics in the NP, morphological formation of the future tense.
^,^ 8As an anonymous reviewer aptly points out, while the loss of the infinitive across Balkan languages may be an effect of contact, lumping together still extant infinitives of other IE languages should not be done without first testing these extant infinitives for cognacy. Here, we highlight the use of subjunctive clauses in lieu of infinitives, and while we agree with the reviewer that several of these features still need reworking, in the current application of this method we are not capitalizing on the complement of the set of languages that have lost their infinitive.


**Table 3:** Strong positive Pearson Correlation Coefficients for the Balkan Sprachbund.

Character	Description (WALS chapter)	PCC
124A2	Subject is expressed overtly (‘Want’ Complement Subjects)	0.90
102A3	Both the A and P arguments (Verbal Person Marking)	0.76
104A3	P precedes A (Order of Person Markers on the Verb)	0.64
23A1	Head marking (Locus of Marking in the Clause)	0.61
104A4	Both orders of A and P occur (Order of Person Markers on the Verb)	0.61
37A3	Definite affix (Definite Articles)	0.53
71A4	Special imperative + special negative (The Prohibitive)	0.49

Among the characters listed for the Balkan group, one can find characters that need to be reworked and become more theoretically informed: Languages that mark both A and P arguments (following WALS sampling procedures and feature definitions) may do so either by subject and object agreement (e.g. Kashmiri, Persian) or by subject agreement and object clitic doubling (Albanian, Greek, Romanian, Bulgarian, Macedonian). A more fine-grained definition and sampling procedure would disentangle these two distinct linguistic phenomena resulting in characters with either stronger phylogenetic signal (a character targeting verbal agreement only) or sensitive to contact (a character targeting clitic doubling).

Another way we analyzed the data was to compare the r value per character for nodes representing genera, when the respective old languages are included and when they are not (method (b)). This would provide a list with more conservative characters. For all language groups but Italic, including the older languages in the sample resulted in more characters with absolute correlation values above the |r| = 0.3 threshold. The correlation values between a genus and a character also gets higher if the respective old language is included in the group. Again Italic is an exception to that, and the characters that correlate with it have a lower absolute |r| value if Latin is included in the language group: this suggests that all the characters that are typical of (the) Romance (languages in our sample) are not shared with their common ancestor – this is not a surprising finding if we take into consideration the radical changes in the evolution of Latin (Ledgeway 2012) and especially the fact that Classical Latin is not the immediate common ancestor of the modern Romance languages, which arguably sprang out of local varieties of Vulgar Latin which underwent parallel changes. Such changes (such as the emergence of a definite article, pronominal clitics, loss of morphological case distinctions etc.) followed unidirectional historical pathways that historical linguists commonly treat as ‘drift’ (in Sapir’s 1921 sense).

Third (method (c)), we divided language groups between those that are comprised of languages with a common ancestor (apart from Proto-Indo-European, PIE) and those that are comprised of languages that are close geographically; we then identified the minimum and maximum correlation values for each class of language groups per character: a character that has high absolute correlation values against at least some historical language group is deemed to carry phylogenetic signal, while a character that has high absolute correlation values against at least some geographical language group is sensitive to changes induced by language contact. Thus, the correlation values are used to create a ‘profile’ for each character based on whether it correlates with language groups that belong to the same genus/sub-genus and/or with language groups that do not belong in the same branch but share traits due to (extensive) language contact. This shows whether the character carries phylogenetic signal and/or is susceptible to changes due to language contact. For example, Table 4A shows the correlation values obtained when comparing character 37A1 with each group. Table 4B shows the minimum and maximum value per class of language groups for the same character, 37A1. Character 37A1 seems to be important for an IE phylogeny since it attains relatively strong correlations (r > 0.30 or r < −0.30; marked in bold in Tables 4A and 4B) with many historical groups of IE and it does not seem to be sensitive to horizontal transfers (−0.30 < r <0.30).

**Table 4A:** Example of the correlation values (r) between a character (37A1) and all language groups tested. For |r| ≥ 0.3 boldface is used.

Language group	Character 37A1: Definite word distinct from demonstrative (Definite Articles)
**Italic**	**0.38**
**Romance**	**0.45**
**Celtic**	**0.47**
**Balto-Slavic**	**−0.42**
**Modern Balto-Slavic**	**−0.39**
**Slavic**	**−0.37**
**Baltic**	−0.14
**Modern Slavic**	**−0.35**
**Germanic**	0.03
**West Germanic**	**0.4**
**North Germanic**	−0.2
**Modern Germanic**	0.11
**Indo-Iranian**	−**0,33**
**Modern Indo-Iranian**	−**0,3**
**Indic**	−0.26
**Iranian**	−0.14
**Modern Indic**	−0.23
**Albanian**	−0.1
**Armenian**	−0.1
**Greek**	**0.32**
**Modern Greek**	0.23
**Balkan**	−0.08
**Albanian-Romanian**	−0.14

We profiled each character this way. The results of all characters can be found in the Supplementary material (‘Bipartitions_Pearson_correlations’). Table 5 shows a few representative examples of characters with strong phylogenetic signal that seem not to have been affected by horizontal transfers, at least in IE. Table 6 on the other hand shows cases of characters that are characteristic of the Balkan Sprachbund (a geographical group). The characters are plotted along with the highest correlation values they get when correlated with historical groups.

**Table 4B:** ‘Profile’ of a character based on its correlation values (r) with historical (H) and geographical groups (G). For |r| ≥ 0.3 boldface is used.

Minimum and maximum r value per group type	37A1: Definite word distinct from demonstrative (Definite Articles)
min-H	−**0.42**
max-H	**0.47**
min-G	−0.14
max-G	−0.08

**Table 5:** Example characters with phylogenetic signal based on correlation values. For historical groups, only the ones with |r| > 0.3 are reported.

Character	Historical language group (correlation value)	Geographical group (correlation value)
**62A5: Action nominal constructions – Other**	North Germanic (1.00), Modern Germanic (0.73), Germanic (0.67)	Balkan (−0.11)
**144J1: Word & NoDoubleNeg (SVNegO)**	North Germanic (1.00), Modern Germanic (0.73), Germanic (0.67)	Balkan (−0.11)
**82A2: Order of Subject and Verb: VS**	Celtic (1.00)	Balkan (−0.11)
**88A2: Order of Demonstrative and Noun: Noun-Demonstrative**	Celtic (1.00)	Balkan (−0.11)
**27A2: Reduplication: Full Reduplication Only**	Armenian (1.00)	Balkan (−0.05)
**112A3: Negative Morphemes: Variation between negative word and affix**	Armenian (1.00)	Balkan (−0.05)
**81A1: Order of Subject, Object and Verb: SOV**	Modern Indo-Iranian (0.92), Indo-Iranian (0.86), Modern Indic (0.82), Indic (0.73), Iranian (0.50)	Balkan (−0.15)
**95A1: Relationship between the Order of Object and Verb and the Order of Adposition and Noun Phrase: OV and Postpositions**	Indic (0.91), Indo-Iranian (0.86), Modern Indic (0.82), Modern Indo-Iranian (0.76)	Balkan (−0.15)
**48A2: Person Marking on Adpositions: Pronouns Only**	Celtic (0.88)	Balkan (−0.12)

In Table 6 one can identify three types of characters: first, there are characters that reflect Balkanisms, which do not have a high absolute |r| value for any IE genus, not even for the genera that Balkan languages belong to (124A2, 102A3); these characters do not carry phylogenetic signal. Second, characters that seemingly contain mixed signal, as they have a high absolute |r| value for contact groups (Balkan and/or Albanian-Romanian) as well as for historical groups; however, these historical groups contain languages which also belong to the Sprachbund; e.g. 23A1 and 104A4 reflect states shared by only Bulgarian and Macedonian, increasing the correlation value both for their own genus (Slavic) and for Balkan. So, in such cases it is not clear whether the high correlation value should be attributed to vertical or horizontal transfer. Finally, there are characters that have a high correlation value both for a geographical group and genera unrelated to it, e.g. 37A3 and 23A3. This last group of characters may be a more accurate case of characters carrying mixed signal, as these characters are significant for both geographical and distinct historical groups. However, even such characters need to be qualitatively assessed: for example, 23A3 (marking of the object on the Head and/or the Dependent in the clausal domain) has a ‘Yes/1’ value for Romanian, Spanish, Albanian, Greek, Hindi, Gujarati, Marathi, Kashmiri, and Pashto. The shared distribution seems to result from the fact that this character conflates regular case marking and Differential Object Marking (DOM) as ‘dependent marking’, while also conflating object agreement and clitic doubling as ‘head marking’.9Differential Object Marking (DOM) is a phenomenon where an object only receives marking when it is of a special type, for example only nouns denoting humans receive accusative marking in Spanish. Clitic doubling is a phenomenon where a clitic object pronoun is added to a sentence which already includes an overt NP representing the P argument. If these linguistic phenomena are teased apart, the Sprachbund languages that have double marking as a result of case marking and clitic doubling of the object will form a single group, separate from Indo-Iranian languages that have double marking as a result of object agreement on the verb and case marking/DOM.

**Table 6:** Correlation values |r| > 0.3 for characters that are affected by contact.

Character	Historical language group (correlation value)	Geographical group (correlation value)
**124A2: ‘Want’ Complement Subjects: Subject is expressed overtly**	Albanian (0.38), Modern Greek (0.38)	Balkan (0.90), Albanian-Romanian (0.55)
**102A3: Verbal Person Marking: Both the A and P arguments**	Albanian (0.32), Modern Greek (0.32)	Balkan (0.76), Albanian-Romanian (0.46)
**88A3: Order of Demonstrative and Noun: Mixed**	Italic (0.35), Romance (0.38)	Albanian-Romanian (0.70), Balkan (0.43)
**104A3: Order of Person Markers on the Verb: P precedes A**	Albanian (0.48), Greek (0.31), Modern Greek (0.48), Romance (0.34)	Albanian-Romanian (0.69), Balkan (0.64)
**23A1: Locus of Marking in the Clause: Head marking**	Modern Slavic (0.40), Slavic (0.38), Modern Balto-slavic (0.36), Balto-slavic (0.34)	Balkan (0.61)
**104A4: Order of Person Markers on the Verb: Both orders of A and P occur**	Modern Slavic (0.40), Slavic (0.38), Modern Balto-slavic (0.36), Balto-slavic (0.34)	Balkan (0.61)
**37A3: Definite Articles: Definite affix**	North Germanic (0.63), Modern Germanic (0.40), Germanic (0.31), Albanian (0.30), Armenian (0.30)	Balkan (0.53), Albanian-Romanian (0.43)
**37A4: The prohibitive: Special Imperative + Special Negative**	Modern Greek (0.38)	Balkan (0.49)
**23A3: Locus of Marking in the Clause Double marking**	Modern Indo-Iranian (0.50), Indo-Iranian (0.45), Modern Indic (0.36), Albanian (0.30), Modern Greek (0.30), Balto-slavic (−0.31)	Albanian-Romanian (0.43), Balkan (0.36)
**25A2: Locus of Marking: Whole-language typology Double-marking**	Greek (0.70), Modern Greek (1.00)	Balkan (0.43)
**43A4: Third Person Pronouns and Demonstratives – Related to non-remote demonstratives**	Greek (0.70), Modern Greek (1.00)	Balkan (0.43)
**144A1: Position of Negative Word With Respect to Subject, Object, and Verb – SNegVO**	Modern Slavic (0.49), Slavic (0.44), Modern Balto-slavic (0.40), Balto-slavic (0.36), Romance (0.33), Indo-Iranian (−0.31)	Balkan (0.40), Albanian-Romanian (0.34)
**144D1: The Position of Negative Morphemes in SVO Languages – SNegVO**	Modern Slavic (0.49), Slavic (0.44), Modern Balto-slavic (0.40), Balto-slavic (0.36), Romance (0.33), Indo-Iranian (−0.31)	Balkan (0.40), Albanian-Romanian (0.34)

In sum, this method allowed us to identify characters that are typical of language groups that are monophyletic or comprised of languages that are geographically close. First, we identified a threshold above (or in the case of negative correlations below) which the character-states reflected known properties of the language group (method (a)). By examining genus-level groups both with and without their old languages (method (b)), we confirmed that the characters identified in the first step (method (a)) carry phylogenetic signal, as absolute correlation values were higher if old languages were included; Italic and Romance was the only exception to that. Finally (method (c)), we profiled each character based on whether they reflect inherited and/or contact-induced properties.

## 4 Mutations

For the second out of three methods we developed, we made use of a well-known phylogenetic method: calculating parsimony scores. This method needs a reference phylogenetic tree, in order to map the different states of the characters on to it. We built a reference Indo-European (IE) tree using cognate data from Bouckaert et al. (2012), which represents the accepted genealogical history of the external nodes (i.e. languages).10We ran a Bayesian phylogenetic inference using BEAST v2.6.3 (Bouckaert et al. 2014) under a relaxed exponential clock model; Birth Death Skyline Contemporary BDSParam model as prior; as a prior candidate for birth (λ) and death (μ) rate we selected an exponential distribution, mean value 0.01 starting from 0.01 and 0.009 respectively. As the prior candidate for the sampling parameter (ρ) we selected beta distribution (*α* = 54, *β* = 455). We set no constraints for phylogeny. We ran the model for 100,000,000 generations sampling every 1,000 generations with a burn-in of the first 1,000 samples; we generated the maximum clade credibility (MCC) tree using TreeAnnotator v2.6.3 after discarding the first 50 % of the trees. We then mapped each of our 425 binary characters onto the languages of the tree and calculated the minimum number of mutations needed to get the (known) distribution of the character states across the languages. This is the parsimony score of the character. A full set of these mutation mappings can be found in the Supplementary material.11The tree diagrams in the supplementary material (‘Parsimony_scores_for_IE’), a few of which are presented below, are not rooted; this is because the parsimony score of any tree, even a rooted one, is not dependent on where it is rooted.
^,^ 12Features for which either (a) all languages share the same state, or (b) all but one language share the same state, have not been plotted.


Low parsimony scores mean that we need fewer mutations to get the final distribution of the character and therefore this distribution agrees with the tree. High parsimony scores mean that many independent mutations are needed to explain the distribution of the characters given the tree and therefore, this character does not agree with the topology of the tree. We then used the parsimony score and the locus of the mutations to assess the characters in two steps: first, to evaluate how accurately the mutations reflect known historical events of language change; second, to assess the phylogenetic information contained in them.

**Figure 1:**
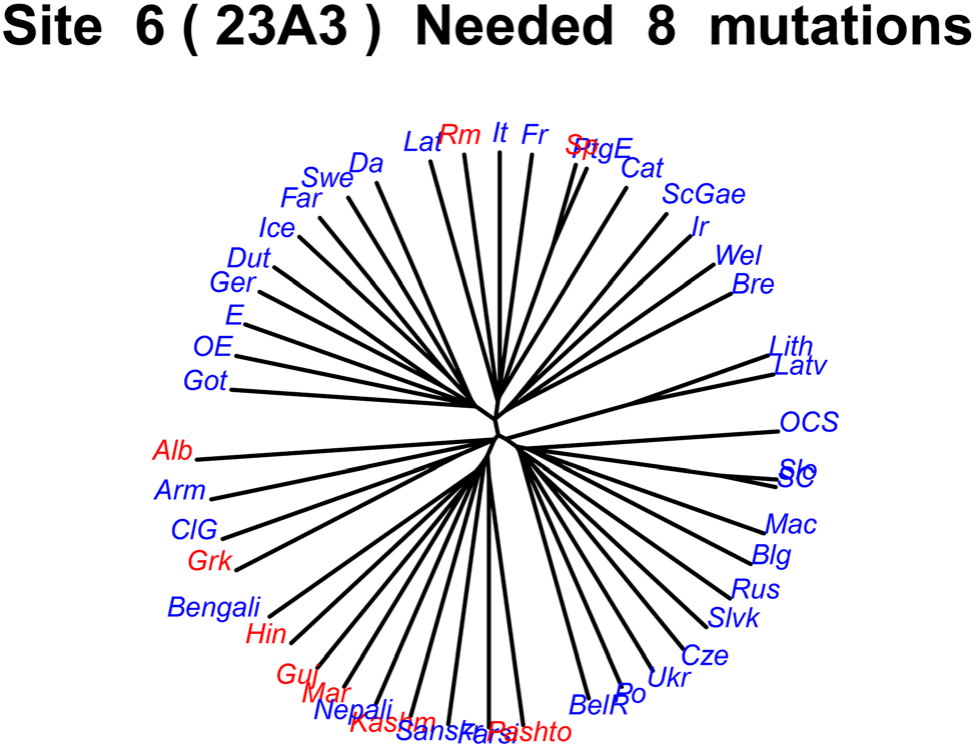
The schema represents the IE tree. The distribution of states for character 23A3 *Marking of the Patient argument on the head and the dependent (double marking)* is represented by the font color of the language (1/Yes = red, 0/No = blue). On top is the parsimony score of the character (8 mutations).

Consider the tree in Figure 1, which represents the IE phylogeny marked for character 23A3 *Marking of the P(atient) argument on the Head and the Dependent (double marking)*. Branches with the same color share the same state: red represents the state ‘1/Yes’, blue represents state ‘0/No’; in our case, red represents languages with double marking, both on the head (the inflected verb) and on the object noun phrase (NP), while blue represents languages without double marking of the patient (they may have no marking at all, only dependent marking, or only head marking).

Assuming that a trait can be present or absent, and is inherited along the tree, the *mutations* method calculates how often the state of the character changed from absent to present or from present to absent. In principle, it could be the case that the Proto-language had either state. If we consider that blue was the initial state, then we expect four independent mutations/innovations: Spanish, Romanian, Albanian and Greek. For the case of Indo-Iranian there are multiple mathematically equivalent scenarios: either there was an innovation at the common ancestor of Indo-Iranian (1) and it was then (independently) lost by Nepali, Sanskrit, and Farsi (3); alternatively, it was innovated by Pashto and independently by the Indic branch after splitting from Sanskrit (2), after which Bengali and Nepali lost it (2). Either scenario involves 4 mutations; adding these to the 4 we already had, gives a total of 8.

However, if we look closer at the actual data, as already discussed above in the section on the bipartitions method, this double marking is actually a result of the interplay between 4 distinct phenomena: Clitic Doubling of the object, Verbal agreement with the object, case marking of the object, and DOM. The Indic branch is indeed characterized by verbal agreement with the P argument. Greek, Albanian and Romanian on the other hand exhibit clitic doubling (see footnote 9 above). According to WALS chapter 23, clitic doubling is counted as head marking, on a par with agreement of the P argument on the verb. From the perspective of formal syntactic theory, however, grouping clitic doubling, a phenomenon which essentially repeats the P argument as a phonologically weak pronoun that happens to attach to the verb, together with verbal agreement through an affix on the verb, is not supported by any deeper theoretical connection between clitic doubling and verbal agreement mechanisms. The similarity between these phenomena is superficial, leading to an incorrect proliferation of mutations and calling for revision of the relevant characters. In a similar fashion, case marking of the dependent and DOM are treated alike by the WALS definition, which was also adopted in our database. If, however, the character was theoretically informed and did not count DOM as an instance of case marking, then Spanish and Pashto, which exhibit DOM but do not otherwise have case marking on nouns, would also be blue, and thus the number of mutations would be reduced by two.

After establishing in the first step that the mutations needed are genuine, i.e. they do not reflect some grouping of phenomena that is not theoretically supported, we proceeded with the second step of the assessment of the phylogenetic information contained in the character. We divided this step into three possible outcomes: First, a character can be considered a marker for either the whole family or for a specific branch of the tree (genus/sub-family, or a branching below that level) if most of the languages on that branch display the same character state. Second, it can be assumed to be horizontally transferred if it appears in non-closely related languages in the tree, which are known to have been in contact. Third, it can be homoplastic if it has arisen independently in different languages (e.g. Differential Object Marking occurring independently in Romance and in Indic).

Consider the example *Noun*-*Adjective order* in Romance and Celtic (character 87A2). Noun-Adjective (N-Adj) order is shared among all Romance languages as well as all Celtic languages, so it is a marker of their respective genera; this order is derived when the noun undergoes movement across the adjectives either to a site just below D (Romance) or to D itself (other genera; see Cinque 1994: 87).13One could possibly argue for a feature that marks the Italo-Celtic clade.


**Figure 2:**
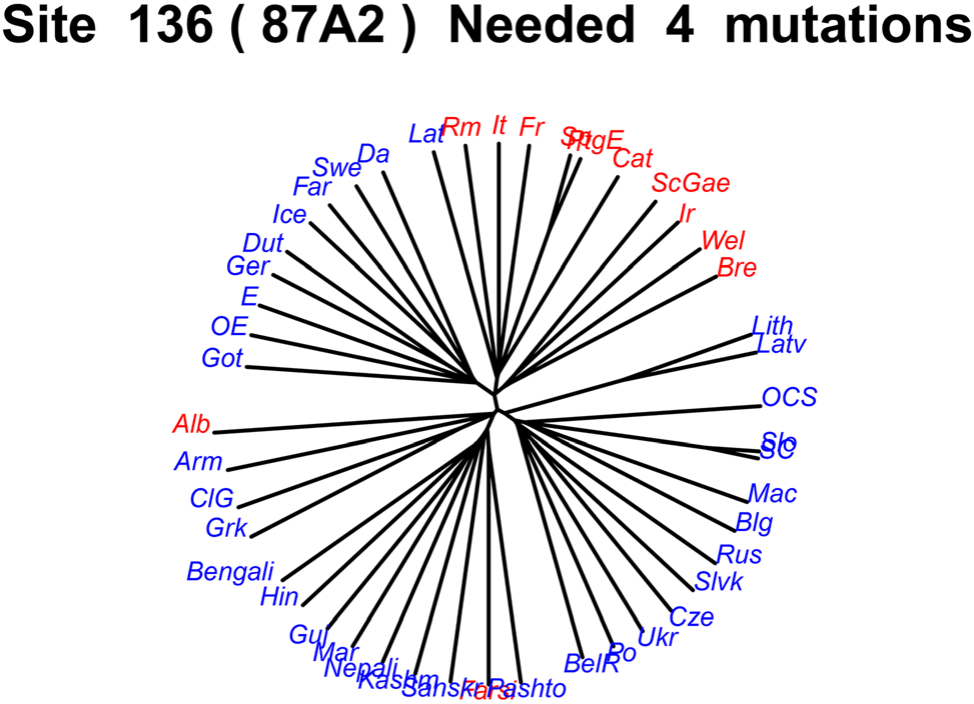
Parsimony score for character 87A2 *N-Adj word order* in IE. Languages in red have N-Adj, whereas languages in blue have other states (Adj-N/mixed). On top is the parsimony score of the character (4 mutations).

A different case is the N-Adj order in Albanian and Farsi, which require the use of linkers when adding adjectives to an NP, reversing the expected order (den Dikken 2006; Taghipour and Kehnemuyipour 2023).14Thus, this is another instance of splitting distinct linguistic phenomena, as we have seen above for case marking/DOM and verbal agreement/clitic doubling for WALS chapter 23A. So at the level of IE, Ν-Αdj order associated with the presence of linkers is a homoplastic character, occurring in two otherwise unrelated branches independently. Thus, even with a low parsimony score (4 mutations, see Figure 2), multiple unrelated phenomena may still be conflated.

Turning back to our feature scrutinizing procedure based on mutations, a third outcome is that a character can be assumed to be horizontally transferred if it appears in non-closely related languages in the tree which are known to have been in contact (e.g. lack of infinitives in the Balkan languages). This is reflected in character 124A2 (also identified by the bipartition method above), as languages with infinitives usually cannot express the subject overtly; the Balkan languages have lost infinitives and use a subjunctive clause instead.

While this method is useful to pinpoint where morphosyntactic characters need refinement, it has also showed us that we are not yet at the stage where we can single out characters that encode purely phylogenetic, purely areal, or purely any other type of signal, as the same character may at once serve as a marker of one branch, while also showing up in other branches due to contact or homoplasy. For example, strict VSO characterizes Celtic as a genus marker in IE but it also characterizes Afroasiatic languages, presumably serving as a family marker. This common pattern is *homoplastic*. A feature can even be a genus marker while occurring as a homoplasy in a different branch due to contact with a language external to the family; or be spread horizontally between genetically related languages which also happen to be geographically close. Disambiguating between these factors is not a trivial undertaking, and more advanced methods need to be developed to do so.

## 5 Hill-climbing approach to identify informative subsets of features

The third and final method we developed is a variant of the classic hill-climbing algorithm for optimization. Our objective was to find the subset(s) of characters out of the larger pool of characters that would produce a phylogeny as close as possible to the target phylogeny.

For this method, we built a target IE tree, using Bayesian inference, of the languages in our sample, using the cognate data for those languages from Bouckaert et al. (2012).15Just like the target tree used for the mutations method, we built a target tree using cognate data by means of Bayesian phylogenetic inference with BEAST v2.6.3 (Bouckaert et al. 2014) under a relaxed exponential clock model. The tree can be found in the supplementary material (‘Reference_tree_IE2011’).
^,^ 16Here it must be noted, firstly, that the topology of our target tree, while based on the cognacy dataset IELex, previously used by Bouckaert et al. (2012), diverges from that of their summary tree on several points; and, secondly, that both trees diverge on several points from received wisdom about the history of IE. We thank the anonymous reviewer who highlighted these issues. One reason why our tree diverges from that of Bouckaert et al. (2012) is simply that we used a subset of their language sample. While the reality of building phylogenetic trees using Bayesian inference is that changing the language sample or a few parameters can make a large difference in the outcome of the tree, using the same method (Bayesian inference) was the best way to build a baseline tree for the purposes of this particular experiment. We would furthermore like to stress that the actual topology of the tree makes little difference to the experiment per se: honing in on the subset of characters that can best replicate a target tree only needs the target tree to remain constant. For future purposes, determining the best possible target tree for a particular language family will be necessary, as we get closer to finding the subset of characters to build that target tree. The hill-climbing method then sought to reproduce this target tree as closely as possible. The algorithm starts off with a random subset of sites from the larger data pool and builds a phylogenetic tree with just those data. This tree is then compared to the target tree.

In the next step, the algorithm proposes, at random, a small change from the initial subset of sites, and builds a new phylogenetic tree. This second tree is, again, compared to the target tree. If the new tree scores better in terms of approaching the target tree than the previous result, the new subset of data is retained, and the process of proposing a small change of subset at random starts anew. If the new tree does not score better, it is discarded, and the algorithm reverts back to the previous subset and tries again. A systematic overview of the steps is given in (1):

(1)

**Table:** 

Step 1: Propose a small change on the subset of sites
Tree 1 is further from the target → DISCARD the subset
Step 2: Propose a small change on the subset of sites
Tree 2 is closer to the target → KEEP subset 2
Step 3: Propose a small change on subset 2
Tree 3 is further than Tree 2 → DISCARD the subset (keep subset 2)

The tree closest to the target tree was constructed with just 41 features out of the original 425. This subset of 41 features is given in Table 7.

**Table 7:** List of features selected by the algorithm that resulted in the tree closest to the reference tree.

Selected features	WALS feature	WALS value	WALS value
20A1	20A: Fusion of Selected Inflectional Formatives	1	Exclusively concatenative
20A4	20A: Fusion of Selected Inflectional Formatives	4	Tonal/isolating
21A1	21A: Exponence of Selected Inflectional Formatives	1	Monoexponential case
21A2	21A: Exponence of Selected Inflectional Formatives	2	Case + number
21A5	21A: Exponence of Selected Inflectional Formatives	3	Case + referentiality
26A1	26A: Prefixing vs. Suffixing in Inflectional Morphology	2	Strongly suffixing
26A4	26A: Prefixing vs. Suffixing in Inflectional Morphology	1	Little affixation
26A6	26A: Prefixing vs. Suffixing in Inflectional Morphology	6	Strong prefixing
27A3	27A: Reduplication	3	No productive reduplication
28A2	28A: Case Syncretism	3	Core and non-core
28A3	28A: Case Syncretism	4	No syncretism
30A2	30A: Number of Genders	2	Two
30A5	30A: Number of Genders	5	Five or more
33A1	33A: Coding of Nominal Plurality	6	Mixed morphological plural
34A2	34A: Occurrence of Nominal Plurality	6	All nouns, always obligatory
37A4	37A: Definite Articles	4	No definite, but indefinite article
38A2	38A: Indefinite Articles	2	Indefinite word same as ‘one’
39A4	39A: Inclusive/Exclusive Distinction in Independent Pronouns	2	‘We’ the same as ‘I’
40A2	40A: Inclusive/Exclusive Distinction in Verbal Inflection	2	‘We’ the same as ‘I’
41A3	41A: Distance Contrasts in Demonstratives	3	Three-way contrast
43A2	43A: Third Person Pronouns and Demonstratives	2	Related for all demonstratives
43A6	43A: Third Person Pronouns and Demonstratives	6	Related for non-human reference
44A6	44A: Gender Distinctions in Independent Personal Pronouns	4	1st or 2nd person but not 3rd
47A1	47A: Intensifiers and Reflexive Pronouns	1	Identical
48A1	48A: Person Marking on Adpositions	2	No person marking
49A4	49A: Number of Cases	4	4 cases
50A5	50A: Asymmetrical Case-Marking	6	Syncretism in relevant NP-types
52A1	52A: Comitatives and Instrumentals	1	Identity
52A3	52A: Comitatives and Instrumentals	3	Mixed
57A4	57A: Position of Pronominal Possessive Affixes	1	Possessive prefixes
58A2	58A: Obligatory Possessive Inflection	1	Exists
60A3	60A: Genitives, Adjectives and Relative Clauses	3	Genitives and relative clauses collapsed
64A3	64A: Nominal and Verbal Conjunction	3	Both expressed by juxtaposition
65A2	65A: Perfective/Imperfective Aspect	2	No Grammatical Marking
67A1	67A: The Future Tense	1	Inflectional future exists
67A2	67A: The Future Tense	2	No inflectional future
69A3	69A: Position of Tense-Aspect Affixes	5	No tense-aspect inflection
71A4	71A: The Prohibitive	4	Special imperative + special negative
73A2	73A: The Optative	2	Inflectional optative absent
74A3	74A: Situational Possibility	3	Other kinds of markers
75A1	75A: Epistemic Possibility	1	Verbal constructions

While this method seems promising, it needs more work. Whereas in the other two methods we could provide a qualitative analysis of the distribution of features, for this method, such an interpretation of the data is more elusive. One of the reasons for this is that hill-climbing algorithms can be notoriously hard to converge. This list of characters is only one of the possible outcomes of this method. Every time the algorithm runs, it generates a different set of characters. The algorithm needs to be run many more times in order to identify a stable list of recurring features. What is also worth noting is that in 14 out of these 41 characters all languages of our sample share the same state. The hill-climbing method of taking subsets of our morphosyntactic characters does show us two things: it shows us (i) that there is phylogenetic signal in syntax, and (ii) that modulo finding the most optimal subset of characters in future work, the number of characters need not be enormous; one can replicate a tree built off cognacy characters with a relatively small number of morphosyntactic characters.

## 6 Conclusion

To conclude, we believe that using morphosyntactic data as an alternative to cognate data has the potential to create phylogenetic trees that reach beyond our existing knowledge of proto-languages. However, to our knowledge, no attempts have been made to formulate a list of suitable morphosyntactic characters to apply to languages, similarly to the curation that led to the Swadesh lists for cognate data. A central question to curate morphosyntactic characters for phylogenetic research is: How can we determine which common traits are inherited and which are the result of contact, or are homoplasies?

In this paper, we took a pool of WALS-based typological characters and tested the morphosyntactic data in three ways: We calculated the Pearson correlation coefficient for each character relative to several different language groupings in an attempt to differentiate between historical and areal effects on language change. We compared the parsimony scores of each feature to a target tree to help evaluate superficially similar phenomena grouped together by WALS. Lastly, we administered a hill-climbing approach to attempt to build a phylogeny with subsets of the pool of data, as close as possible to an IE reference tree.

We found that all three methods gave an insight into the data and were helpful tools for the much needed scrutiny of morphosyntactic characters, but that additional linguistic and historical knowledge is necessary to properly evaluate the characters. Both the correlations as well as the mutations method uncovered characters that without modification group superficially similar phenomena together, even though they are distinct according to the theoretical linguistic literature, e.g. object clitics and object agreement, adjective noun word order with and without linkers, case marking and DOM.

The hill-climbing approach is a promising method to aid in discovering subsets of characters which may together produce the best tree – this seems a fruitful approach because given the nature of languages and linguistics, we should not expect islands of independent features to make a meaningful difference; rather, multiple features are expected to form groups and covary. It is important to work out these interdependencies between morphosyntactic characters, because in Bayesian probabilistic tree building methods, the characters are assumed to be independent.

However, we must conclude that extrapolation and carry-over of our findings into other language families is not trivial: there seems to be no intrinsic diffusibility of features, i.e. one cannot claim that some features have greater propensity to transfer than others regardless of their geographical and historical setting (on this, see also Wichmann and Holman 2009 for a quantitative study). Further research is needed to determine how and if we may disentangle horizontal from vertical transfer. We expect that making the definitions of the features more theoretically informed will produce a stronger signal; in order to verify this, the methods should be rerun with updated characters in future work.

## Supplementary Material

The supplementary material can be viewed via the following link: https://drive.google.com/drive/folders/1mD62pkcuWpxkrJbx8uU6Lt-mFuMaRx45?usp=sharing.
